# Real-time fault detection for IIoT facilities using GA-Att-LSTM based on edge-cloud collaboration

**DOI:** 10.3389/fnbot.2024.1499703

**Published:** 2024-11-11

**Authors:** Jiuling Dong, Zehui Li, Yuanshuo Zheng, Jingtang Luo, Min Zhang, Xiaolong Yang

**Affiliations:** ^1^School of Computer and Communication Engineering, University of Science and Technology Beijing, Beijing, China; ^2^School of Information Science and Technology, Hainan Normal University, Haikou, China; ^3^State Grid Sichuan Economic Research Institute, Chengdu, China

**Keywords:** internet of things, fault detection, edge-cloud collaboration, attention mechanism, LSTM

## Abstract

With the rapid development of Industrial Internet of Things (IIoT) technology, various IIoT devices are generating large amounts of industrial sensor data that are spatiotemporally correlated and heterogeneous from multi-source and multi-domain. This poses a challenge to current detection algorithms. Therefore, this paper proposes an improved long short-term memory (LSTM) neural network model based on the genetic algorithm, attention mechanism and edge-cloud collaboration (GA-Att-LSTM) framework is proposed to detect anomalies of IIoT facilities. Firstly, an edge-cloud collaboration framework is established to real-time process a large amount of sensor data at the edge node in real time, which reduces the time of uploading sensor data to the cloud platform. Secondly, to overcome the problem of insufficient attention to important features in the input sequence in traditional LSTM algorithms, we introduce an attention mechanism to adaptively adjust the weights of important features in the model. Meanwhile, a genetic algorithm optimized hyperparameters of the LSTM neural network is proposed to transform anomaly detection into a classification problem and effectively extract the correlation of time-series data, which improves the recognition rate of fault detection. Finally, the proposed method has been evaluated on a publicly available fault database. The results indicate an accuracy of 99.6%, an F1-score of 84.2%, a precision of 89.8%, and a recall of 77.6%, all of which exceed the performance of five traditional machine learning methods.

## 1 Introduction

With the widespread application of artificial intelligence and Internet of Things technologies in Industry 4.0, Industrial Internet of Things (IIoT) technology greatly improves and optimizes the operational and production efficiency of industrial equipment while reducing enterprises’ human resource costs (Liu et al., 2023; Zhang et al., 2024; Feng et al., 2022). However, IIoT technology also increases the complexity of production equipment. As a result, the large amount of sensor data generated raises the probability of equipment failure. Additionally, industrial equipment is influenced by the external environment and its own harsh operating conditions during actual industrial production. Therefore, the sensor data exhibits spatio-temporal correlations and high-dimensional characteristics, such as bearing wear data, motor condition data, and air pressure, humidity, and temperature data from aircraft in the aerospace sector (Wang et al., 2021; Xu et al., 2022). This complexity poses significant challenges to traditional fault detection techniques (Zhang et al., 2023; Aboelwafa et al., 2020; Akgüller et al., 2024). Consequently, accurate and timely detection of abnormal phenomena is crucial for ensuring the safety and efficient operation of industrial equipment. Currently, fault detection research methods can be classified into three main categories:

Univariate and multivariate probability statistical methods are utilized based on the characteristics of equipment data. A single index, such as the mean, variance, and peak, is commonly used for fault detection in single-feature equipment sensor data. Wang et al. (2022) proposed a fault detection method for wind turbine blades based on the transmissibility function of wavelet packet energy, which enhanced high-frequency resolution while maintaining its low sensitivity to noise. Zhang et al. (2022) adopted L2-norm shapelet dictionary learning to improve the bearing fault recognition rate under uncertain working conditions. Meanwhile, Peng et al. (2021) realized wind turbine fault detection based on fault characteristic frequency recognition by using compressive-sensing-based signal reconstruction technology and signal reconstruction analysis. Additionally, Shi et al. (2022) designed a generalized variable-step multiscale Lempel-Ziv algorithm to extract features of rolling bearings. The univariate fault detection method is simple and efficient, but performs poorly in identifying equipment failures caused by multiple factors. To provide a comprehensive analysis of equipment operation, a statistical method based on multivariate fault detection is proposed. Lei et al. (2021) proposed Hertz contact theory to detect faults in angular contact ball bearings by taking into account the influence of centrifugal force, thermal impact on bearing operation, and gyroscopic moments. Bhatnagar et al. (2022) used the discrete wavelet transform to obtain discriminative features of fault current signals for detecting faults in distribution networks. This study can effectively identify common shunt faults and high-impedance faults in distribution lines. Multivariate fault detection methods can provide a comprehensive view of equipment status. However, the overall fault detection rate may decrease in the presence of numerous missing sample data and complex high-dimensional scenarios.

An equipment fault detection method based on spatial distance and region. Wang (2018) mentioned that the fault in nonlinear processes can be detected by the modified conventional kernel partial least squares method, which has definitely improved the computing speed. To overcome the limitations of the principal component analysis algorithm, Shah et al. (2023) proposed a manifold learning method based on the weighted linear local tangent space alignment to provide local tangent space estimates under the condition that uniformly distributed data is not close to linear subspaces. Qin et al. (2022) used a combination of correlative statistical analysis and the sliding window technique for diagnosing initial faults, which improved the recognition rate and reduced the computational complexity. Zhang et al. (2021) proposed an SR-RKPCA model based on subspace reconstruction for detecting wind turbine faults. Compared with traditional principal component analysis and KPCA methods, this approach can better extract nonlinear features of wind turbine data. Sarmadi and Karamodin (2020) worked on removing the environmental variability conditions and estimating local covariance matrices to find sufficient nearest neighbors for training and testing datasets in a two-stage procedure. The study used adaptive Mahalanobis-squared distance and one-class KNN algorithms to classify the fault patterns. Wang et al. (2021) considered relevant hidden information in the temporal dynamics of frequencies and spatial configuration for training a K-nearest neighbor classifier based on a temporal-spatio graph to improve fault diagnosis performance. Distance-based fault detection methods are straightforward, yet their computational time increases rapidly with large-scale and high-dimensional fault data, rendering them unsuitable for real-time detection in industrial settings.

A fault detection method based on machine learning. To enhance the intelligence and efficiency of fault detection, some scholars have applied machine learning technology to the field of fault detection and have achieved certain results. In their study, Sun and Yu (2022) proposed an innovative adaptive technique based on sparse representation and minimum entropy deconvolution for identifying bearing faults, which promoted the effectiveness of impulse enhancement and the robustness of the inverse filter length. To overcome the problem of significant noise interference in bearing vibration signals, Chen et al. (2023) extracted the signal features by using a hierarchical improved envelope spectrum entropy method and identified the bearing faults using a support vector machine. Dhibi et al. (2020) proposed a reduced kernel random forest method to address the limitations of a single random forest algorithm in industrial processes and applied it to the fault detection of grid-tied photovoltaic systems.

Machine learning methods transform fault detection into classification problems, which offers the advantages of short training times and strong generalization abilities. Nonetheless, significant noise pollution can lead to suboptimal fault detection rates. Therefore, Xue et al. (2022) proposed a stacked long short-term memory (LSTM) network to enhance the performance of fault diagnosis. However, the hyperparameters of the LSTM network are mostly obtained through experience (Zhi et al., 2022). Unreasonable allocation of important feature weights and hyperparameter settings directly impact the fault classification results. Furthermore, the IIoT data are characterized by large scale, multi-source heterogeneity, and high noise, which brings many difficulties and challenges to cloud-based IIoT systems. The challenges include processing real-time data, managing core network loads, maintaining user data security, and ensuring system scalability. To address the aforementioned problems, this article proposes and implements a fault detection model based on the LSTM model, the genetic algorithm, the attention mechanism, and edge-cloud collaboration (GA-Att-LSTM) framework. The major contributions of the article are summarized as follows:

To improve detection speed and reduce the pressure on cloud storage, we utilize an edge-cloud collaborative framework to lower more sensor data computation and storage from the “core” to the “edge,” which have high storage, efficient processing speed, and strong multi-source heterogeneous adaptability.

To extract key temporal features of sensor data, achieve intelligent fault detection, and reduce manual intervention, we use Att-LSTM network to transform complex fault detection problems into classification problems, which has enhanced detection efficiency and decreases equipment maintenance costs.

To obtain appropriate hyperparameters of the LSTM network, we use an improved genetic algorithm (GA) to optimize Att-LSTM network, which has improved the efficiency of fault detection.

The remainder of the article is described as follows: Section2 introduces the architecture principle of edge-cloud collaborative including intelligent terminal layer, edge node layer and cloud platform layer; Section 3 illustrates the methodologies, LSTM structure and GA-Att-LSTM network structure; Section 4 introduces the fault detection principle and design; Section 5 discusses the performance evaluation of data preprocessing and result analysis; Finally, contributions of this article are summarized in Section 6.

## 2 Architecture principle and design

**Figure 1:**
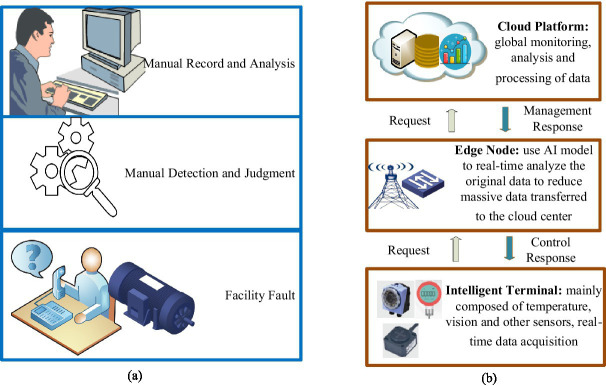
Elucidation of traditional model and state-of-the-art system for facility fault-detection in IIoT. (a) Traditional manual fault detection model; (b) the advanced edge-cloud collaboration fault detection model.

In traditional manual fault detection under IIoT facilities, the operating status of the facilities usually needs to be manually detected, recorded, analyzed, and judged. This method is inefficient, leading to higher maintenance costs and inaccessible, non-real-time results (Huang et al., 2020a). Therefore, the demand for intelligent facility fault detection without human intervention is urgent in Industry 4.0. A facility fault detection model based on cloud-only computing provides some advantages and plays a crucial role in IIoT. Storing data on the cloud server allows a centralized operations facility to monitor systems and process information from various regions and databases (Li et al., 2024a). In cloud-only computing, the delay problem cannot be solved merely by increasing the speed of data transmission without limit (Fu et al., 2018; Li et al., 2024b). To effectively alleviate the latency issue, the distance data must travel needs to be shortened as much as possible. This is why edge computing is used in IIoT. In response to the above problems, this article proposes a model based on edge-cloud collaboration for facility fault detection. The traditional detection model is shown in Figure 1a, while Figure 1b illustrates how the arrangement operates via edge-cloud collaboration.

**Figure 2:**
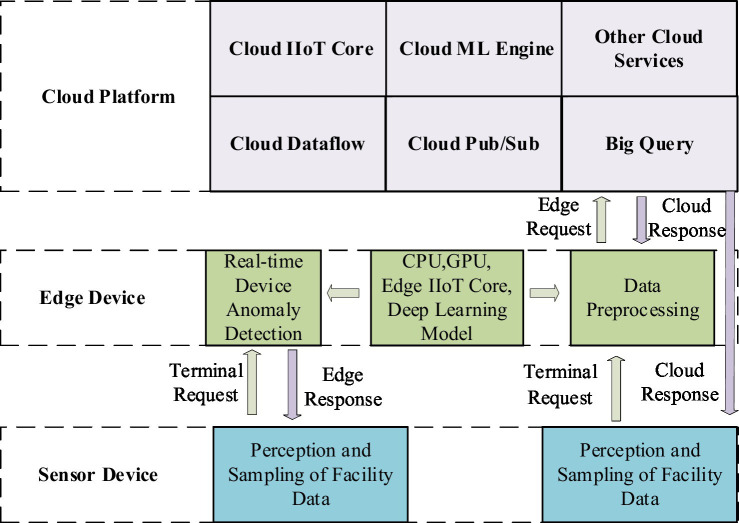
The role of edge nodes in the proposed overall architecture.

As shown in Figure 1b, a fault detection framework based on edge-cloud collaboration is composed of three layers. The intelligent terminal layer comprises the industrial infrastructure, where sensors and industrial facilities are installed. The edge layer is deployed to process collected data in real time. The cloud platform layer is used to train GA-Att-LSTM network models and save weight parameters. The collaboration between the edge and the cloud, along with various sensors and devices, is demonstrated as follows:

1. Intelligent terminal layer: the intelligent terminal layer is the most basic component of a typical edge-cloud-based infrastructure for collecting information. It is mainly composed of sensors, radio-frequency identification, GPS, and cameras (Li et al., 2023a). First, real-time heterogeneous data are primarily obtained using cameras and sensors (for position, speed, energy consumption, pressure, temperature, etc.). Sensors employ a process to convert various signals into electrical signals, which are then processed by related equipment (Kaur et al., 2022; Kaur and Chanak, 2023; Liu et al., 2021). The data are ultimately transmitted to the upper layer using various transmission technologies, such as industrial fieldbus, industrial Ethernet, industrial wireless networks, Bluetooth, and infrared.
2. Edge node layer: the edge node layer is the middle part of the system, mainly composed of gateways and computing nodes (e.g., mobile phones, computers, servers). Gateways provide both visibility and control over connected devices that use the same IIoT protocol. Moreover, they standardize the codec for control commands and device data, after which they transmit the information to the upper layer. This approach avoids the problem of disparate data from multiple collection devices in the cloud (Li et al., 2023b). The computing node layer consists of various nodes through which facility data passes from the gateway to the cloud. During the system’s initialization phase, it acts as a relay device, transmitting the environmental monitoring data collected by wireless sensor nodes to the cloud platform (Yu et al., 2023; Song et al., 2023; Natesha and Guddeti, 2021). Fault detection is performed on the collected data during the system’s routine operation phase. When an abnormal situation is detected, the edge computing node reports the issue to the data and control center on the cloud platform. Simultaneously, it prompts the controller at the bottom layer to offer an emergency response plan. Figure 2 shows the role of the edge computing nodes.
3. Cloud platform layer: the cloud platform layer sits at the top of the architecture, providing significant advantages and influencing the IIoT. The cloud computing platform offers exceptional computational power and large storage capacity, serving as a remote data and control center for the system. This enables a centralized operations facility to monitor systems and optimize parameters for artificial intelligence algorithms (Bui et al., 2020). It is primarily used for processing, storing, and analyzing large-scale global historical data with complex computational requirements. In this article, the edge-cloud collaboration framework is applied to fault detection in equipment to improve maintenance efficiency and leverage the strengths of both technologies. To achieve real-time functionality, edge computing mainly handles short-term, localized data. The LSTM network is an artificial neural model that requires complex parameter training for feature extraction. The computational demands and resource consumption associated with this complexity are challenging for both wireless sensor nodes and edge computing nodes. To address this issue, model training is performed on a cloud-based platform. Real-time fault detection is then carried out by sending the trained model parameters back to the edge computing node.

## 3 Methodology

In this section, we introduce the methodology for developing the edge-cloud collaboration framework for IIoT systems. First, we briefly review Recurrent Neural Networks (RNN) and LSTM models, which are essential for building the proposed GA-Att-LSTM framework. This is followed by a discussion of the system architecture and model development. Finally, we introduce the framework for optimizing the LSTM network using a GA.

### 3.1 Basic recurrent neural network

**Figure 3:**
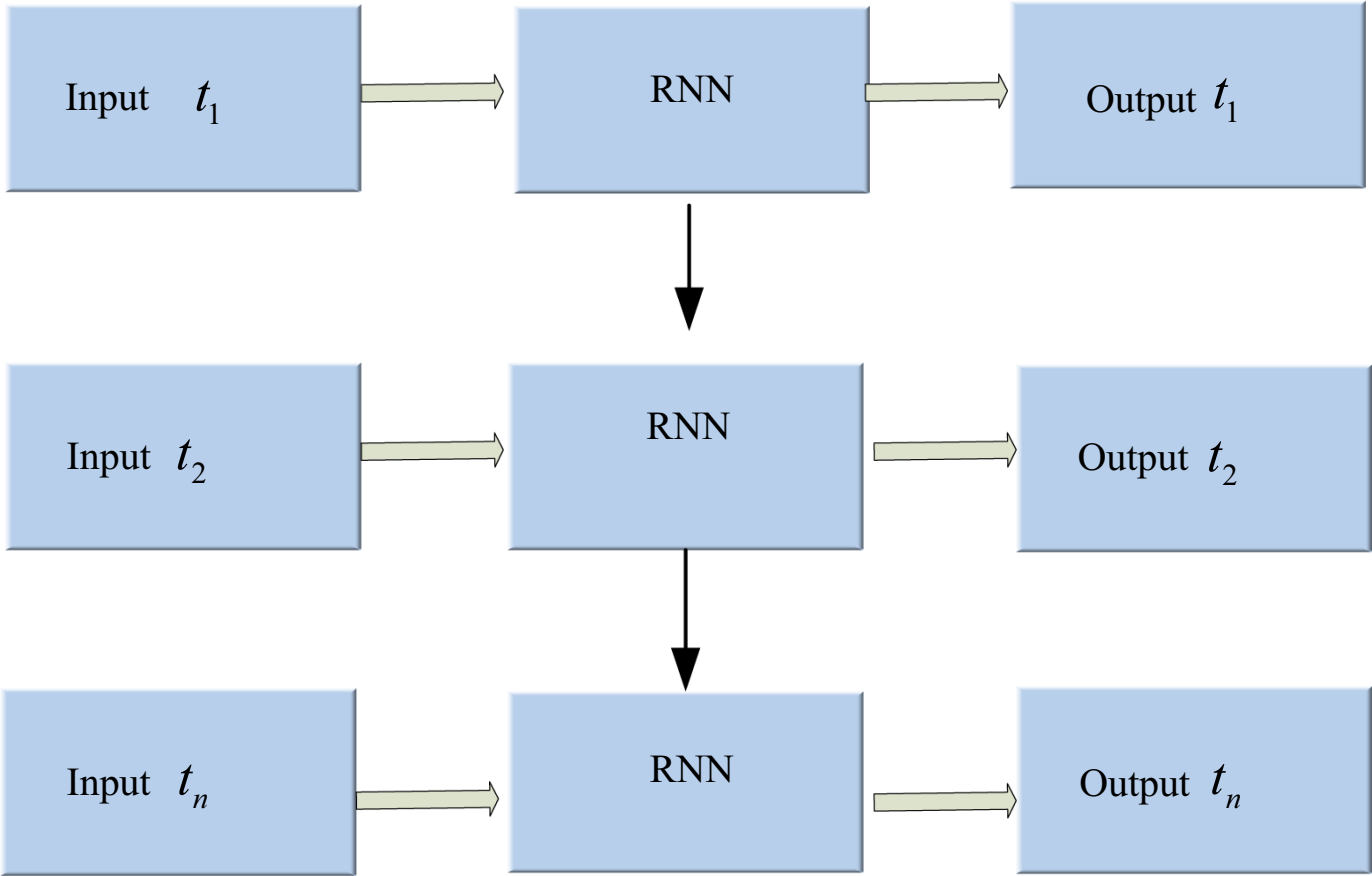
Architecture of recurrent neural network.

The RNN is an architecture with a memory function that stores the previous network operation’s state value and leverages it to generate input for the current moment. It stores the previous network operation’s generated state value and utilizes it to generate the present moment’s input value, enabling RNN to handle time-series sensor data (Abdul et al., 2020). Figure 3 shows the RNN architecture.

**Figure 4:**
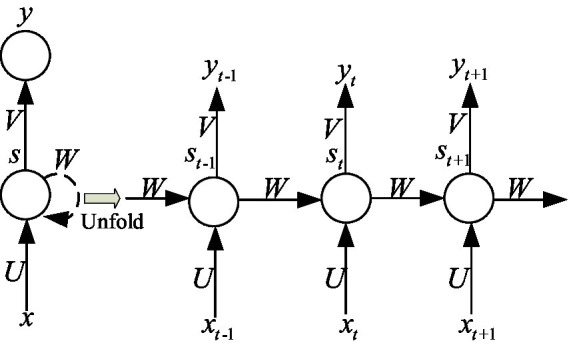
The hidden layer of RNN is expanded according to the time axis.

In Figure 3, the hidden layer blocks are unfolded along the timeline as shown in Figure 4, and their nodes are connected to the corresponding weights through directed loops. Where x is the input vector, s represents the hidden layer vector, y denotes the output vector, weight matrix from the hidden layer to the output layer is defined as U , weight matrix from the hidden layer to the output layer is defined as V and W is the connection weight between the hidden layer cells.

In IIoT systems, the input values at different time steps are denoted as $x_{t-1}$ , $x_{t}$ and $x_{t+1}$ , where each represents the input at a specific time step in a sequence. The input $x_{t-1}$ at time step t−1 represents the value immediately preceding the current input. The input $x_{t}$ at time step t is combined with the previous hidden state to update the current hidden state. The input $x_{t+1}$ at time step t+1 is used as the network advances through the sequence. $x_{t}$ , $s_{t}$ , and $y_{t}$ represents input value, memory value, and output value at time step t respectively. The value of $s_{t}$ is related to the $x_{t}$ at current moments and the $s_{t-1}$ at the previous time. These internal relationships between the input, hidden, and output layers are expressed as shown in Equations (1, 2):


(1) $$s_{t}=f\left(Ux_{t}+Ws_{t-1}\right)$$

(2) $$y_{t}=g\left(Vs_{t}\right)$$

where $g\left(\cdot \right)$ and $f\left(\cdot \right)$ denote activation functions, respectively. From the given (1)–(2), it is clear that the weights are indicative of the dependence relationship between input values at time step t and t−1 . Thus, they are commonly used in many sequence learning tasks. However, as the time series grows, the initial gradient contribution diminishes and the chain of gradients lengthens, resulting in gradient vanishing. To address this issue, the LSTM network is proposed.

### 3.2 Long short-term memory model

**Figure 5:**
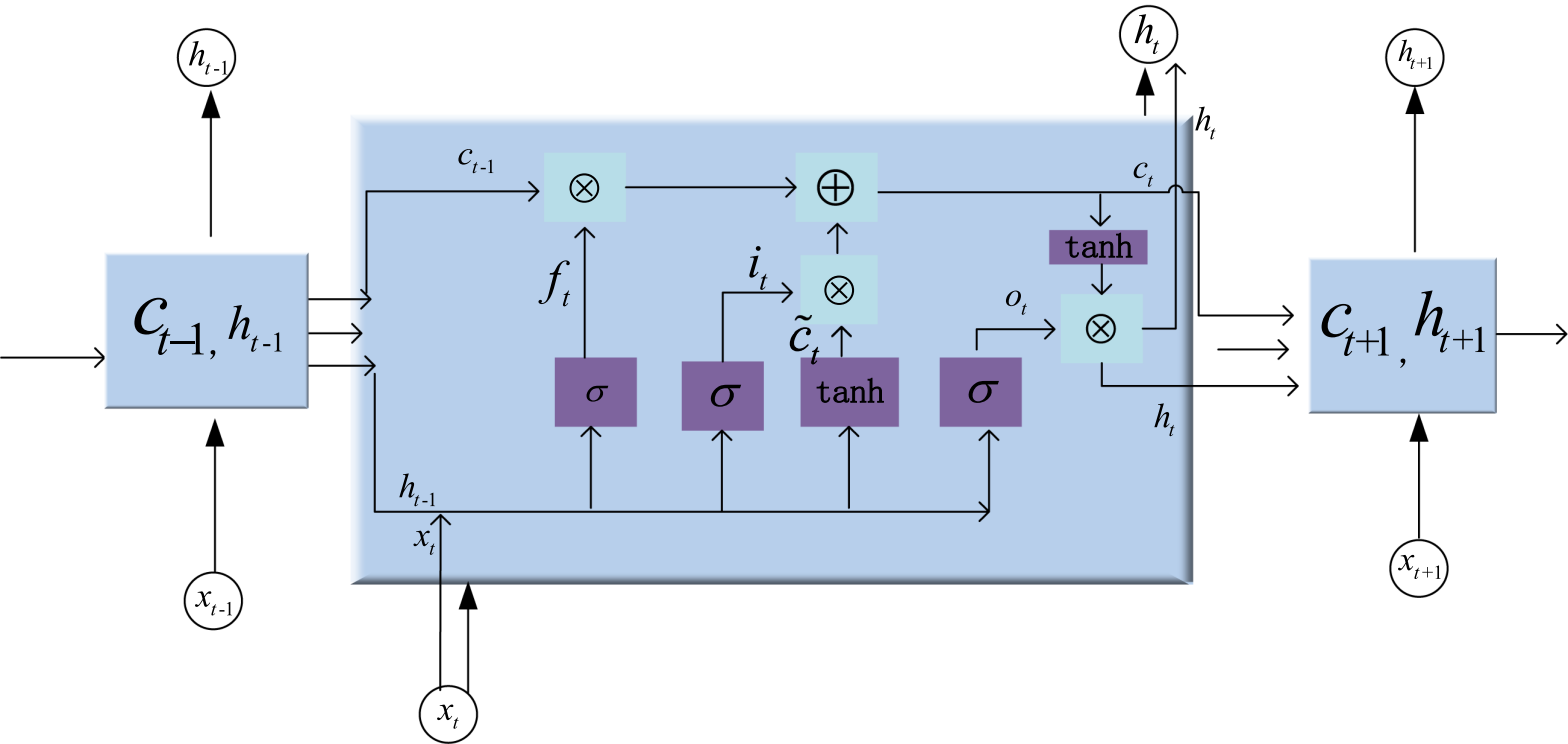
Internal structure of LSTM block.

The LSTM network can solve the problem of vanishing or exploding gradients that exists in ordinary RNN by designing input gates ( $i_{t}$ ), forget gates ( $f_{t}$ ), and output gates ( $o_{t}$ ) (Huang et al., 2024; Lin and Zhang, 2024). Where $c_{t}$ stands for the long-term memory unit, ⊙ symbol represents the multiplication of the corresponding elements. $\sigma \left(x\right)$ denotes the non-linear sigmoid activation function with the value range from 0 to 1, which is used to describe the number of information passing through. W and b are the weight matrices and bias terms, respectively. $x_{t}$ represents the input vector, the short-term state is $h_{t}$ . The unit structure of hidden layer is shown in Figure 5. Since LSTM has a memory block and gate structure, it can learn information with a long span and determine the optimal time lag autonomously. When processed time series data are fed into the LSTM network, the forgetting gate first determines which information needs to be discarded. An input vector $x_{t}$ and a previous short-term state $h_{t-1}$ are utilized for inputs to the forget gate. The output value is calculated using the sigmoid function. The range is 0 to 1. A value of 0 implies that information may pass through while 1 implies the opposite. After passing through the input gate, the relevant information is selected for storage in the cell state. The sigmoid layer determines which values should be updated, while the tanh layer generates a new candidate value vector and calculates the new cell state. Lastly, the output gate decides which information to output. The current cell state is processed by tanh and multiplied by the sigmoid layer’s output to produce the final output.

The input gate decides the amount of information flows from the input *x_t_* that is retained in the cell state *c_t_* at the present time. The output vector *i_t_* of the input gate is given by He et al. (2023) as shown in Equation (3).


(3) $$i_{t}=\sigma \left(W_{\mathrm{xi}}x_{t}+W_{hi}h_{t-1}+b_{i}\right)$$

The forget gate has the function that saves partial information flow of the previous moment in the cell state $c_{t-1}$ to the current moment $c_{t}$ . The candidate cell state ${\tilde{c}}_{t}$ is a crucial element in LSTM that serves as a proposed update to the existing cell state. It is based on both the current input and the past hidden state. The output of the forget gate $f_{t}$ and the memory cell $c_{t}$ at time t are defined as shown in Equations (4–6).


(4) $$f_{t}=\sigma \left(W_{xf}x_{t}+W_{hf}h_{t-1}+b_{f}\right)$$

(5) $${\tilde{c}}_{t}=\tanh\left(W_{xc}x_{t}+W_{hc}h_{t-1}+b_{c}\right)$$

(6) $$c_{t}=f_{t}⊙c_{t-1}+i_{t}⊙{\tilde{c}}_{t}$$

The output gate in Equation (7) mainly controls the influence of long-term state $c_{t}$ on the current short-term state $h_{t}$ , i.e., the data in $c_{t}$ will be output at time t . The output of the output gate $o_{t}$ and output value of short-term state $h_{t}$ in Equation (8) are given as follows:


(7) $$o_{t}=\sigma \left(W_{xo}x_{t}+W_{ho}h_{t-1}+b_{o}\right)$$

(8) $$h_{t}=o_{t}⊙\tanh\left(c_{t}\right)$$

When training LSTM network model, it’s common to use a loss function to evaluate the error between prediction and actual values. The smaller the loss function, the better the performance of the model. To measure the degree of difference between two probability distributions in the same random variable, we use the cross-entropy loss function in Equation (9) for measurement. Its expression is derived as follows:


(9) $$J\left(\theta \right)=-\frac{1}{N}\overset{N}{\underset{i=1}{\sum }}y_{i}\times \ln{\hat{y}}_{i}$$

where N represents the number of samples, $y_{i}$ is the real value of samples, and ${\hat{y}}_{i}$ stands for the predicted value of samples. Firstly, Adam algorithm is used as an optimizer to update the weight of the neural network model, which is simple to implement, computationally efficient and low memory requirement. Then, the loss function is used to calculate the error of each iteration. Finally, the trained neural network model is used to predict the results.

### 3.3 Attention mechanism

**Figure 6:**
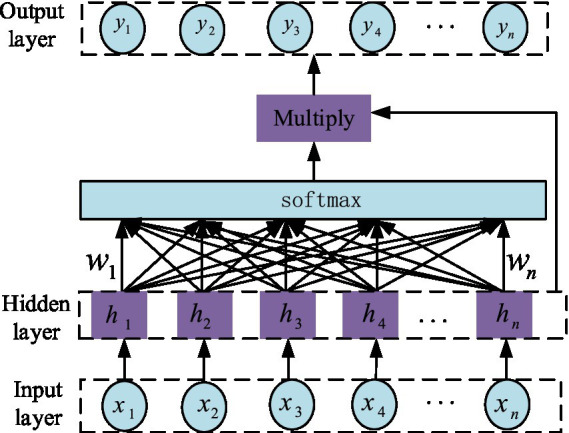
Internal structure of Attention mechanism.

The attention mechanism model, jointly proposed by Treisman and Gelade, aims to mimic human attention and is particularly suitable for optimizing the performance of traditional models. The core function of the attention mechanism is to calculate and analyze the data features input into the model, assigning corresponding probability weights to each feature in the neural network’s hidden layer based on the analysis results. In this process, more important features receive higher weights, thereby improving the output accuracy of the network model (Yuan et al., 2021). The structure of the attention mechanism is shown in Figure 6. The variables $x_{1},x_{2},x_{3}\cdots x_{n}$ represent the input sequences, the variables $h_{1},h_{2},h_{3}\cdots h_{n}$ represent the hidden sequences, and $y_{1},y_{2},y_{3}\cdots y_{n}$ are the output sequences. $w_{n}$ is the attention weight.

### 3.4 GA-Att-LSTM model

**Figure 7:**
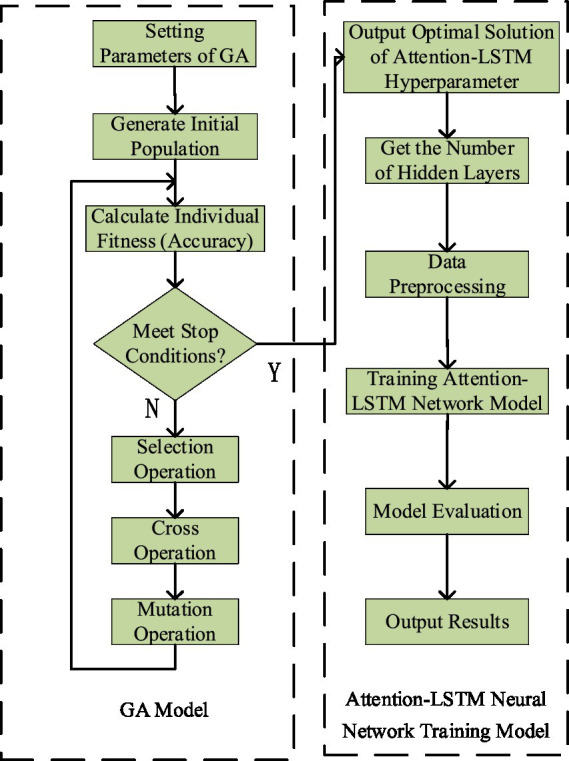
Flow chart for optimizing attention-LSTM network with GA.

The GA is a highly efficient, parallel, and adaptive global probabilistic search method that mimics the process of biological evolution and inheritance in natural environments. By using GA to optimize the number of layers and neurons in each layer of an LSTM network, the architecture selection process can be automated, significantly reducing the complexity of manual tuning. The algorithm continuously generates, evaluates, and selects new architecture candidates by simulating natural selection and genetic mechanisms. Through crossover and mutation of high-fit individuals, it creates increasingly diverse network structures, gradually eliminating less effective models while refining both the number of layers and neuron allocation. As iterations progress, the GA effectively explores the parameter space and ultimately identifies the optimal LSTM model for a given task, striking an optimal balance between network complexity and predictive accuracy. The main process of the GA-Att-LSTM model is illustrated in Figure 7.

## 4 Fault detection principle and design

### 4.1 Fault detection with traditional method

Fault detection aims to identify the abnormal data points. In IIoT systems, the irregular data can be detected by analyzing regular sensor data within the spatio-temporal domain. There are many reasons for outlier data, including unexpected events within the monitoring area (e.g., abnormal device shutdown or sudden power failure) and abnormalities within the sensor node itself (e.g., hardware module damage, low node power). Many traditional methods have been exploited to predict the facilities failure (Li et al., 2024c). The fault detection methods commonly used are mainly multinomial naive bayes (MNB) (Bennacer et al., 2014), logistic regression (LR) (Huang et al., 2020b), principal component analysis-recurrent neural Network (PCA-RNN) (Mansouri et al., 2022), k-nearest neighbor (KNN) (Zayed et al., 2023), AdaBoost (Hussain and Zaidi, 2024), and gradient boosting classifier (GBC) (Al-Haddad et al., 2024). Despite their widespread use, these algorithms have significant limitations. For example, MNB assumes independence between features, resulting in reduced classification performance in situations with strong feature correlations or class imbalances. LR, on the other hand, is limited to linear decision boundaries and performs poorly in the presence of complex non-linear relationships unless features are transformed or interaction terms are included. KNN, on the other hand, faces challenges related to high computational complexity, particularly when calculating distances between each sample and all training instances in large datasets, and is sensitive to high dimensionality and noise. AdaBoost is prone to overfitting in noisy environments or unbalanced datasets due to its tendency to continuously increase the weights of misclassified samples. Finally, the GBC is characterized by prolonged training times and high computational complexity, particularly when handling large datasets. It is also susceptible to overfitting if hyperparameters are not adequately optimized, especially in the presence of noisy data. Traditional methods struggle to achieve same-layer capabilities in spatio-temporal problems, mainly due to their inability to connect nodes within the same layer. In contrast, RNN not only learn data features independently, but also allow the current state to receive feedback from the previous state (Li et al., 2021). Given the inherent correlations between asset data points, RNN can detect outliers in asset data more accurately than traditional methods.

### 4.2 Fault detection with GA-Att-LSTM algorithm

#### 4.2.1 Principle of fault detection for edge-cloud collaboration

**Figure 8:**
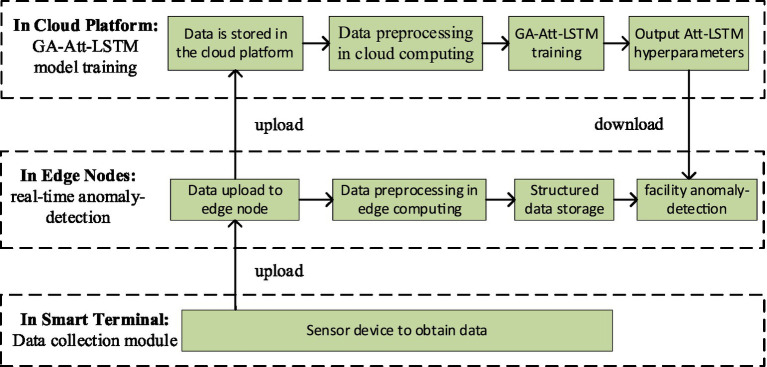
Fault detection process of the GA-Att-LSTM model in IIoT facilities.

In fault detection for IIoT facilities, the GA-Att-LSTM model is proposed. Figure 8 illustrates the calculation process which is primarily divided into three layers: system data acquisition, network model training, and fault detection.

#### 4.2.2 Data acquisition stage

Data acquisition layer establishes connections between the control system, sensor system, system integrated control, and other core nodes in industrial equipment, which mainly rely on industrial ethernet, edge gateways, various sensor devices to communicate with the system. In the process of sensor data acquisition, the data acquisition layer connects the core nodes of industrial equipment such as control systems, sensor systems, and system integration control. These nodes mainly rely on industrial Ethernet, edge gateways, and different kinds of sensor devices to communicate with the system. Therefore, the control system gets operation data of the equipment, which is acquired by the sensor nodes periodically through the network. The data vector generated by node at time t are shown in Equation (10).


(10) $$x_{i}^{t}={\left[x_{i,1}^{t},x_{i,2}^{t},x_{i,3}^{t},\ldots ,x_{i,j}^{t}\right]}^{T}$$

where j is the number of physical variables monitored by node i .

Usually, the sensor data are uploaded to the cloud platform for storage, calculation, and analysis. However, this transmission process takes a long time. As a result, equipment may be damaged due to delayed data transmission. To solve the above problems, we deploy business data that needs to be processed in a timely manner on the edge platform, which can alleviate the huge pressure of massive data on the network bandwidth and satisfy the demand of connected devices for low latency. Further analyzed from a security perspective, the risk of leaking sensitive data during transmission on the public network is avoided because industrial data are stored and analyzed on the edge platform.

#### 4.2.3 Training model hyperparameters in the cloud server service layer

This article utilizes the GA-Att-LSTM model, which is mainly composed of an input layer, a hidden layer and an output layer. During the training phase, the large amount of data consumption requires significant computing resources such as memory, CPU, and hard disk. To mitigate this, training takes place in the cloud service layer. Following this, the trained network parameters (weights, biases, etc.) are passed to the edge computing node, where real-time facility fault detection is performed. Finally, the prediction result is outputted and the relevant response (alarm, shutdown, automatic cooling, etc.) is executed. The historical data stored in the cloud service layer is used as the training data for the model, then the data matrix of sensor node at time is represented as shown in Equation (11):


(11) $$X_{i}=\left[x_{i}^{\left(1\right)},x_{i}^{\left(2\right)},x_{i}^{\left(3\right)},\ldots ,x_{i}^{\left(t-1\right)},x_{i}^{\left(t\right)}\right]$$

#### 4.2.4 Real-time fault detection process in the edge node layer

The computational process of the fault detection model proposed in this article is clearly defined. First, the edge computing system preprocesses the state data collected by sensors from industrial equipment. Next, the GA-Att-LSTM model is employed to assess the abnormality of the equipment. The steps are as follows:

Step 1: Obtain and preprocess sensor data.

Step 2: Split the dataset into training and testing sets using cross-validation.

Step 3: Extract important features from both the training and testing sets.

Step 4: Initialize the parameters of the GA-Att-LSTM network model.

Step 5: Train the GA-Att-LSTM model using the training and testing sets.

Step 6: Output the classification results regarding the operational conditions of the industrial equipment.

## 5 Experiment validation and discussion

### 5.1 Dataset description

To evaluate the efficiency of the proposed GA-Att-LSTM model in IIoT fault detection, we utilize a publicly available machine failure dataset provided by BigML (Huang and Guo, 2019). This dataset consists of 8,784 entries and 28 features, categorized into seven date variables, fifteen numerical variables, and four string variables.

### 5.2 Data preprocessing

**Figure 9:**
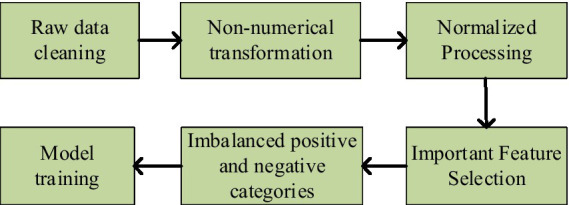
The proposed framework for data preprocessing.

Data preprocessing is crucial in fault detection, as sensor data from equipment may encounter issues such as noise, missing values, inconsistencies, redundant data, and class imbalance. These challenges must be addressed through preprocessing techniques to enhance the accuracy of analysis and prediction. Figure 9 illustrates the framework for data preprocessing.

As shown in the figure above, the data preprocessing process outlines five key steps. Firstly, data cleaning is performed to remove noise and incomplete entries. Next, non-numerical data is transformed to ensure consistency. Next, normalization is applied to enhance data uniformity. Subsequently, important features are selected to improve model performance. Finally, the issue of imbalanced positive and negative categories is addressed to ensure more accurate predictions. The specific steps are detailed as follows:

#### 5.2.1 Data cleaning

Usually we use raw data which may have problems like redundancy, missing, garbled etc. Therefore, we need to perform deletion, averaging, filtering and other measures before using the data.

#### 5.2.2 Non-numerical transformation

One-hot encoding is a technique that transforms discrete features into binary vectors in Euclidean space, enabling classifiers to better process categorical data. By mapping each unique value to a binary representation, such as encoding eight operator values as vectors like [1 0 0 0 0 0 0 0] for operator1, this method enhances feature representation and increases dimensionality.

#### 5.2.3 Normalized processing

The data are normalized, i.e., the eigenvalues of the sample are converted to the same dimension, and the range of values of each feature is mapped uniformly linearly to the interval [0,1]. The normalized formula is shown in Equation (12).


(12) $${\bar{x}}_{i,q}^{\left(t\right)}=\frac{x_{i,q}^{\left(t\right)}-\min\left(x_{i,q}\right)}{\max\left(x_{i,q}\right)-\min\left(x_{i,q}\right)}$$

where $x_{i,q}=\left[x_{i,q}^{\left(1\right)},x_{i,q}^{\left(2\right)},x_{i,q}^{\left(3\right)},\ldots ,x_{i,q}^{\left(t-1\right)},x_{i,q}^{\left(t\right)}\right]$ represents the physical variable q monitored by the sensor node i and the historical data vector stored in time t . $\max\left(x_{i,q}\right)$ and $\min\left(x_{i,q}\right)$ are the maximum and minimum values of $x_{i,q}$ respectively. The optimization process of the optimal solution will obviously become smoother and it will be easier to correctly converge to the optimal solution after the error data cancel the errors caused by different dimensions during training and after the data are normalized.

#### 5.2.4 Important feature selection

**Figure 10:**
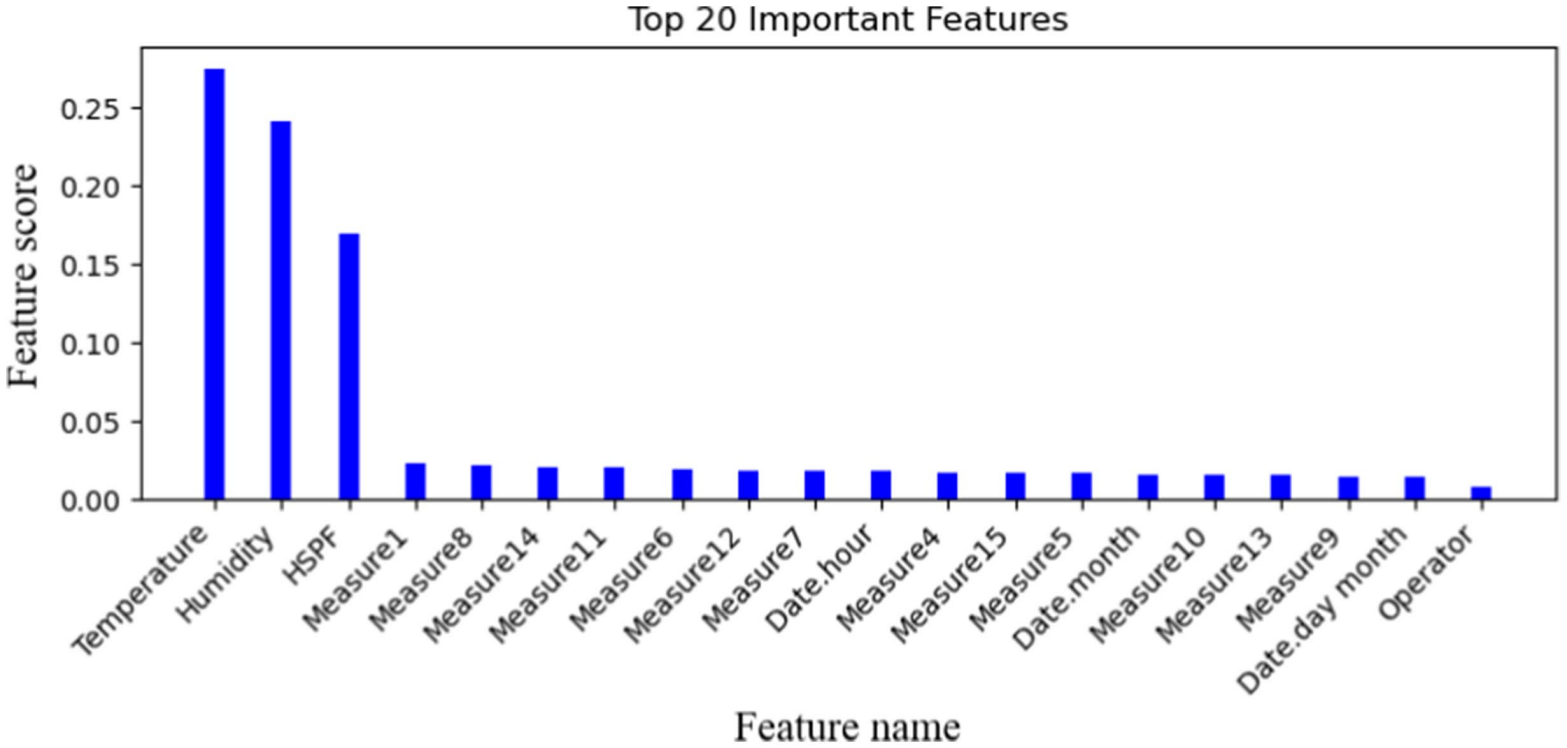
The most important 20-dimensional features proposed.

When the data collected by various sensors involve multiple feature values, not all data’ feature is helpful to the prediction of facility failure. To improve calculation efficiency, this article only selects the 20 important features that are closely related to the equipment operation state by using the random forest classifier method. The important feature values are defined in Figure 10.

#### 5.2.5 Imbalanced positive and negative categories

**Figure 11:**
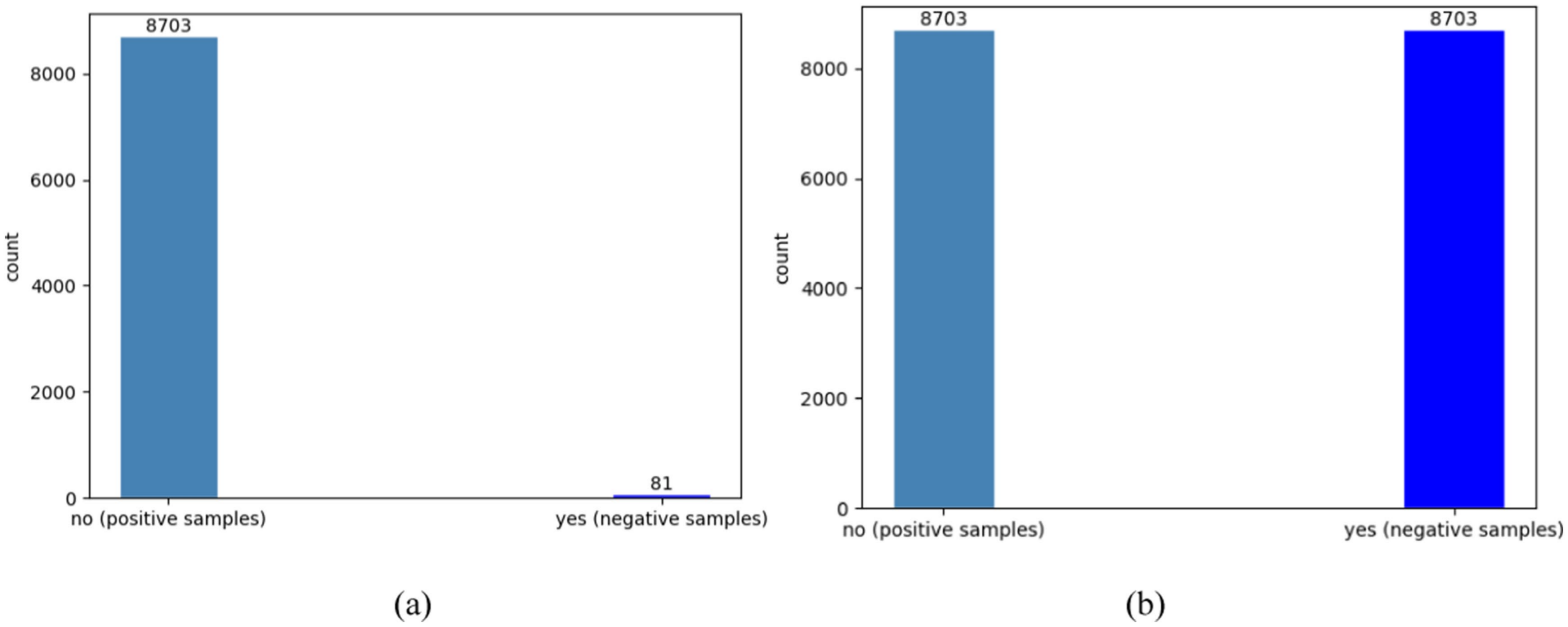
The comparison of positive and negative sample counts before and after optimization using SMOTE. (a) The original number of samples; (b) the number of samples after preprocessing.

The failure feature is utilized as a label and is composed of two values: yes and no. “No” represents the normal operation of the facilities and refers to positive samples, while “yes” indicates that the device is functioning abnormally and refers to negative samples. After conducting a statistical analysis, the dataset shows that the ratio of positive samples to negative samples is around 107:1. It is important to note that the raw dataset is extremely imbalanced since there are significantly more normal records. In particular, we utilized the synthetic minority oversampling technique algorithm (SMOTE) to preprocess the data and balance the number of normal and failure cases. This entailed increasing the number of failure label samples through interpolation to eliminate category imbalances in the training set. Figure 11a depicts the actual ratio of positive and negative samples in the database, while Figure 11b illustrates the ratio of positive and negative samples after preprocessing.

### 5.3 Validation and evaluation of performance

In this paper, common classification metrics are used to evaluate the performance of the fault detection model, including accuracy (Acc) (Ogaili et al., 2024), precision (P) (Wang et al., 2024), and recall (R) (Wang et al., 2024) and F1-score (Li et al., 2023c; Li et al., 2022). Accuracy is the proportion of correctly predicted samples out of the total number, calculated as the sum of true positives (TP) (Guo et al., 2024) and true negatives (TN) (Lee et al., 2024) divided by the sum of TP, TN, false positives (FP) and false negatives (FN) (Sultan et al., 2024). Recall measures the proportion of actual positive samples that are correctly identified by the model, while precision refers to the proportion of predicted positive samples that are actually positive. The F1-score is the harmonic mean of precision and recall, providing a balanced measure of performance, particularly in imbalanced datasets. The respective formulas are as follows:


(13) $$Acc=\frac{TP+TN}{TP+TN+FP+FN}$$

(14) $$P=\frac{TP}{TP+FP}$$

(15) $$R=\frac{TP}{TP+FN}$$

(16) $$F1-\mathrm{score}=\frac{2TP}{2TP+FP+FN}$$

where TP represents correctly predicted positive cases, TN refers to correctly predicted negative cases, FP indicates incorrectly predicted positive cases, and FN represents incorrectly predicted negative cases. These terms correspond to the counts in the confusion matrix and provide a comprehensive assessment of the classifier’s performance in fault detection.

### 5.4 Results and analysis

#### 5.4.1 Performance evaluation of LSTM model with GA

In this paper, the TensorFlow and Keras frameworks are used alongside the GA-Att-LSTM algorithm in the context of device fault detection in the IIoT. The GA-Att-LSTM model is configured with an input layer and output layer of 2 and 20 parameters, respectively, with a learning rate of 0.001 to ensure effective convergence. The number of hidden layers and the number of nodes in each layer are typically determined based on empirical methods, which can result in reduced recognition rates for the LSTM model. To improve the efficiency and accuracy of the model, we use genetic algorithms to optimise key parameters, including the number of hidden layers, the number of neurons per layer, and the configuration of fully connected layers. The optimised parameters for the LSTM model after 100 iterations are shown in Table 1.

**Table 1:** The results of GA-optimized Att-LSTM parameters.

Number of layers	Number of nodes each layer	Number of dense layers	Number of dense layers nodes	Learning rate	Acc
1	14	1	12	0.001	0.981
2	(10, 12)	3	(11, 11, 11)	0.001	0.984
1	12	3	(12, 13, 15)	0.001	0.982
1	12	1	13	0.001	0.987
1	15	3	(12, 13, 12)	0.001	0.987
2	(11, 12)	2	(10, 15)	0.001	0.987
2	(11, 12)	2	(15, 15)	0.001	0.996

Experimental results show that when the GA-Att-LSTM model is configured with two layers of 11 and 12 nodes, respectively, and two fully connected layers of 15 nodes each, a detection accuracy of 99.6% can be achieved. Optimisation by genetic algorithms allows systematic exploration and selection of the best hyperparameters, which significantly improves the efficiency and reliability of fault detection.

#### 5.4.2 Performance evaluation of LSTM model with attention mechanism

The paper experimentally validates the significant enhancement of LSTM model performance achieved by integrating attention mechanisms and genetic algorithms. We conducted a comparative analysis of the PCA-RNN model, the standard LSTM model, and the improved LSTM model with attention mechanisms. The average evaluation results over ten trials are shown in Table 2. Various classification metrics, including accuracy, precision, recall, and F1-score, were employed to comprehensively assess each model’s performance. These metrics provide insights into the strengths and weaknesses of different models in fault detection tasks, offering valuable references for future research.

**Table 2:** Evaluation of different LSTM models.

Model	Acc	*P*	*R*	F1-score
PCA-RNN	0.991	0.762	0.485	0.589
LSTM	0.995	0.837	0.776	0.804
GA-Att-LSTM	0.996	0.898	0.776	0.842

The experimental results indicate that the GA-Att-LSTM model outperforms the other two models, particularly in terms of F1-score. This improvement is primarily attributed to the introduction of the attention mechanism, which enables the model to more effectively identify and focus on key features related to equipment failures. Although the accuracy of all three algorithms is similar, the higher F1-score of GA-Att-LSTM demonstrates its advantage in balancing precision and recall, especially when addressing class imbalance issues. This suggests that the GA-Att-LSTM model can reliably detect equipment failures in practical applications, reducing both false positive and false negative rates, thereby providing significant support for the safety and efficiency of industrial IoT systems.

#### 5.4.3 Performance evaluation of the GA-Att-LSTM against various machine learning models

**Figure 12:**
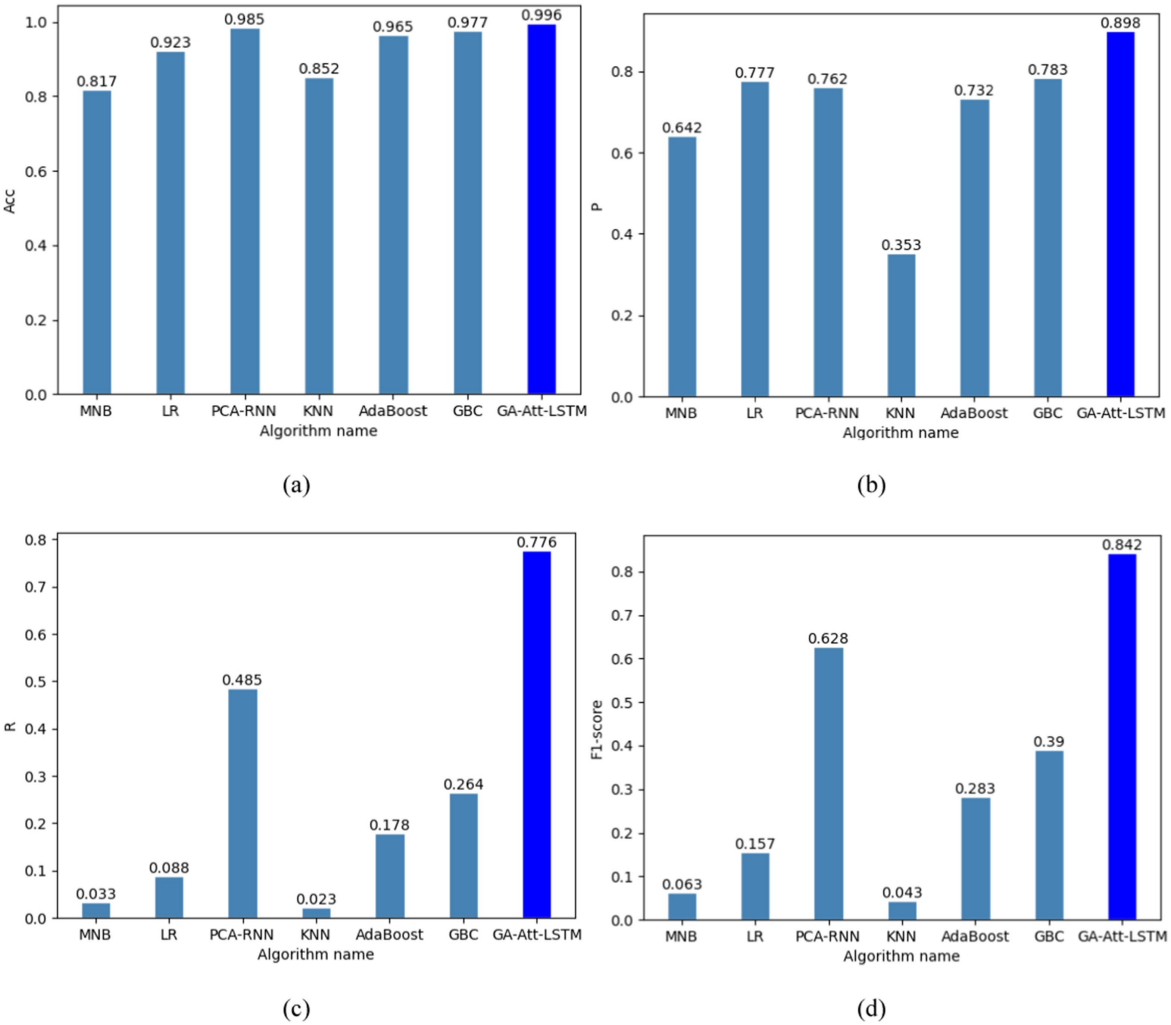
Different evaluation metrics for different models. (a) Accuracy of different models; (b) precision of different models; (c) recall of different models; (d) F1-score of different models.

To further validate the effectiveness of the proposed GA-Att-LSTM model, we compared it with several classical machine learning models, including MNB, LR, KNN, AdaBoost, and GBC. These experiments are designed to systematically assess the performance of different models in fault detection tasks. Based on Equations (13–16), we specifically analyzed the accuracy, precision, recall and F1-score of each model to understand their performance in detecting faults. The comparative results of different algorithms under the same experimental conditions are illustrated in Figure 12.

As shown in Figure 12, the GA-Att-LSTM model achieves an average accuracy of 99.6%, an average precision of 89.8%, an average recall of 77.6% and an average F1-score of 84.2%. These metrics significantly outperform five other machine learning models (MNB, LR, KNN, AdaBoost, GBC) and are slightly higher than those of the PCA-RNN model. This remarkable improvement is mainly attributed to the effective integration of genetic algorithms and attention mechanisms within the GA-Att-LSTM model, which enhances its ability to capture important features and complex relationships in the data, thereby improving prediction accuracy and robustness. Specifically, the GA-Att-LSTM model shows an increase in accuracy ranging from 1.1 to 17.9%, an increase in precision ranging from 11.5 to 54.5%, an increase in recall ranging from 29.1 to 75.3%, and an increase in F1-score ranging from 21.4 to 79.9%. These results indicate that the GA-Att-LSTM model is outstanding in terms of overall performance and balance, thereby improving its generalisation ability. The exceptional performance of the model can largely be attributed to the effectiveness of the LSTM in handling long-term error data received from sensors. In addition, the incorporation of the attention mechanism plays a crucial role in the success of the model. By introducing the attention mechanism between the LSTM and the regression layer, the model processes different input data before applying the attention layer. This mechanism adaptively assigns different weights to the processed data, allowing the model to selectively focus on the most relevant historical sequences, significantly improving classification accuracy.

#### 5.4.4 Performance evaluation of GA-Att-LSTM across different training stages

**Figure 13:**
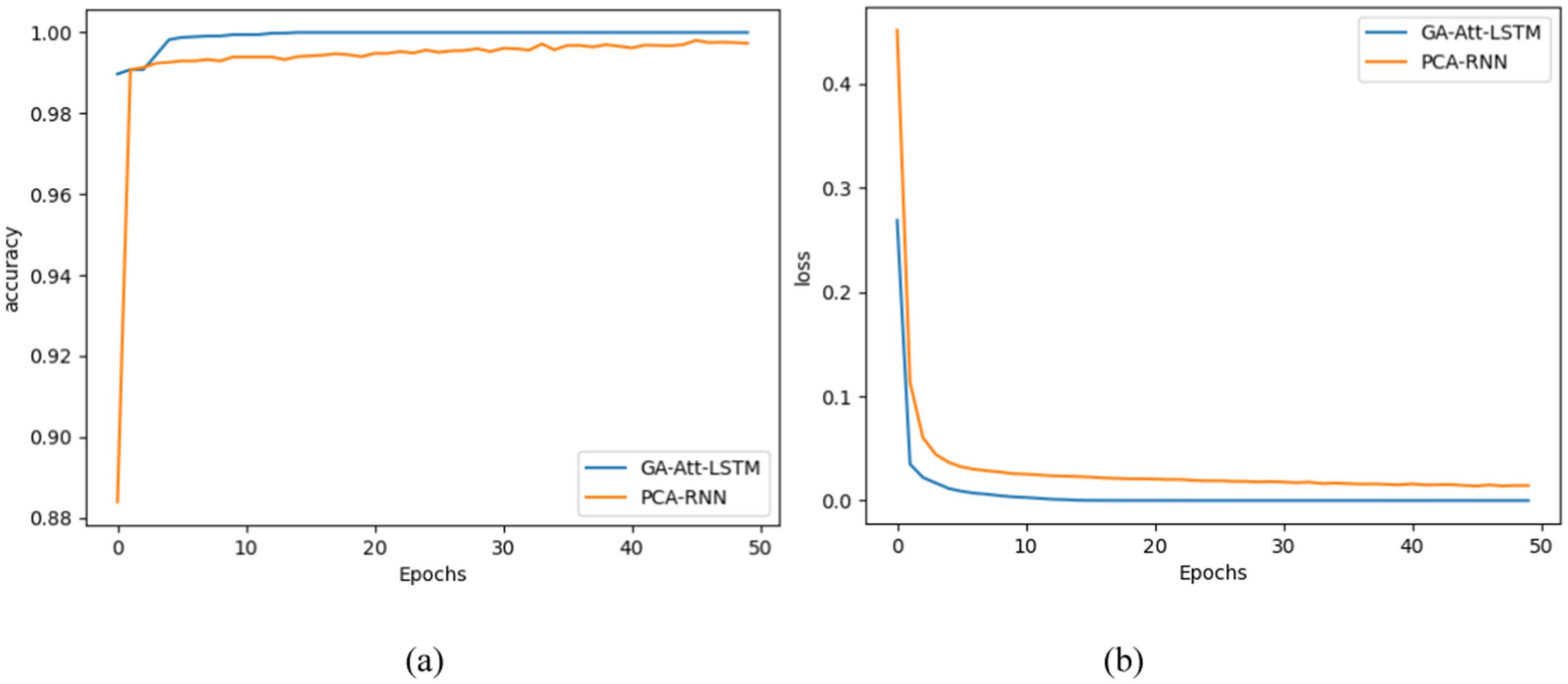
Accuracy and loss curve of GA-Att-LSTM and PCA-RNN in the training stage. (a) Accuracy curve of different network models; (b) loss curve of different network models.

During training of the GA-Att-LSTM model, we introduced regularisation parameters to prevent overfitting and ensure that the model retains good generalisation capabilities when faced with unseen data. Cross-entropy was used as the loss function, which effectively reduced training errors and stabilised learning at each training stage. In addition, we chose accuracy as an evaluation metric to comprehensively assess the model’s performance; this not only reflects the overall predictive ability of the model, but also provides a reference for subsequent optimisation. Figure 13 illustrates the changes in the model’s performance during training, clearly showing trends in training loss and accuracy, which helps to understand the model’s behavior at different stages of training and facilitates further tuning and improvement.

To further compare the learning performance of GA-Att-LSTM model during training stage, accuracy and loss values from different deep learning models are evaluated using iterative curve graphs. In Figure 13, the x-axis represents the number of iterations, and the y-axis represents the accuracy and loss function values for fault identification in IIoT facilities. From Figure 13a, it is evident that as the number of iterations increases, the accuracy of both the GA-Att-LSTM and PCA-RNN models increases, eventually reaching convergence. However, the GA-Att-LSTM model achieves faster convergence and higher final accuracy than the PCA-RNN model. Figure 13b shows that as the number of iterations increases, the loss values of both models decrease until convergence. The GA-Att- LSTM model converges more quickly and achieves a lower final loss value compared to the PCA-RNN model. These results indicate that the proposed method has a stronger feature extraction capability and can quickly learn fault features, leading to faster and more effective model convergence in terms of fault detection.

## 6 Conclusion

This paper presents an edge-cloud collaboration framework for device fault detection using GA-Att-LSTM as the core algorithm. The framework computes a large amount of data from the cloud layer to the edge layer, which improves the multi-source heterogeneous adaptability and reduces the delay. Since traditional LSTM networks cannot focus on the important features in the input sequence at different time steps, this limits their ability and efficiency in processing complex time series data. To address this issue, an attention-based LSTM model is introduced that captures the attention of spatial variables and time samples, and optimises the model hyperparameters using genetic algorithms to improve the detection accuracy. Simulation results show that the GA-Att-LSTM method outperforms six other machine learning algorithms. In future work, we plan to improve the fault detection performance by considering the balance between high accuracy and low time delay in IIoT.

## Data Availability

The datasets presented in this study can be found in online repositories. The names of the repository/repositories and accession number(s) can be found in the article/supplementary material.
